# Hyperoxia in depression

**DOI:** 10.1192/j.eurpsy.2021.1824

**Published:** 2021-08-13

**Authors:** R. Belmaker, Y. Bloch, P. Shvartzman, P. Romem, Y. Bersudsky, A. Azab

**Affiliations:** Faculty Of Health Science, Ben Gurion University of the Negev, Beersheva, Israel

**Keywords:** oxygen, HDRS, CGI, Depression

## Abstract

**Introduction:**

Several studies of normobaric hyperoxia in some neurological conditions have demonstrated clinical benefits. Oxygen enriched air may increase oxygen pressure in brain tissue and have biochemical effects such as on brain erythropoietin gene expression, even in patients without lung disease.

**Objectives:**

This pilot, randomized, double-blind study examined the efficacy of normobaric hyperoxia as a treatment for depression.

**Methods:**

Fifty-five consenting patients aged 18-65 years with mild to moderate depression were included in the study. Participants underwent a psychiatric inclusion assessment and a clinical evaluation by a psychiatric nurse at baseline, 2 and 4 weeks after commencement of study intervention. Participants were randomly assigned to normobaric hyperoxia of 35% fraction of inspired oxygen or 21% fraction of inspired oxygen (room air), through a nasal tube, for 4 weeks, during the night. Patients were rated blindly using the Hamilton Rating Scale for Depression (HRSD); Clinical Global Impression (CGI) questionnaire; Sheehan Disability Scale (SDS).

**Results:**

The present study showed a significant improvement in HRSD (p<0.0001), CGI (p<0.01) and in SDS (p<0.05) among patients with depression who were treated with oxygen-enriched air, as compared to patients who were treated with room air. In CGI, 69% of the patients who were treated with oxygen-enriched air improved compared to 23% patients who were treated with room air.

**Conclusions:**

This small pilot study showed a beneficial effect of normobaric hyperoxia on some symptoms of depression.

**Disclosure:**

No significant relationships.

